# A real-time all-optical interface for dynamic perturbation of neural activity during behavior

**DOI:** 10.1016/j.crmeth.2025.101180

**Published:** 2025-09-18

**Authors:** Zihui Zhang, Patrycja Dzialecka, Lloyd E. Russell, Riccardo Ratto, Christina Buetfering, Oliver M. Gauld, David R. Selviah, Michael Häusser

**Affiliations:** 1 Wolfson Institute for Biomedical Research, University College London, Gower Street, London WC1E 6BT, UK; 2 Department of Electronic and Electrical Engineering, University College London, Torrington Place, London WC1E 7JE, UK; 3 Department of Psychiatry & Behavioral Sciences, Stanford University School of Medicine, Stanford, CA 94305, USA; 4 School of Biomedical Sciences, The University of Hong Kong, Hong Kong, China

**Keywords:** optogenetics, two-photon microscopy, calcium imaging, neural circuits, closed-loop, real-time, behavior, barrel cortex, visual cortex, holographics

## Abstract

We developed a strategy for implementing a dream experiment in systems neuroscience, where circuit manipulation is guided by the real-time readout of neural activity in behaving mice. The system integrates a state-of-the-art calcium imaging analysis package that achieves rapid online activity readout from two-photon calcium imaging, a custom hologram generation program that targets two-photon optogenetic stimulation of specific neuronal ensembles, and software modules that automate essential steps in running complex all-optical experiments. Proof-of-principle experiments demonstrate that neurons can be automatically detected and recruited into a photostimulation ensemble, closed-loop photoinhibition can be implemented immediately after fast mapping of the functional properties of cortical neurons, and targeted activation can be guided by readout of ongoing activity patterns in behaviorally relevant neuronal ensembles during decision-making.

## Highlights

• Holographic optogenetics guided by online analysis of two-photon calcium imaging
• Automatic cell detection and recruitment into a photostimulation ensemble
• Closed-loop activity suppression following fast functional properties mapping
• Targeted neuronal activation guided by ongoing population activity patterns

## Motivation

A fundamental question in neuroscience is how neural activity in the brain drives behavior. Recent advances in optical methods combining two-photon imaging and two-photon optogenetic stimulation have allowed simultaneous readout and manipulation of the activity of individual neurons in awake mice. Such an “all-optical” strategy has allowed neural interventions to be targeted to specific neurons based on their genetic and functional signatures, paving the way to probe their causal role during behavior. However, current all-optical experiments typically activate pre-defined neurons at pre-defined time points, irrespective of the ongoing activity in the local circuit. Since the activity of individual neurons and neural ensembles can be highly variable from trial to trial, it is essential to adjust photostimulation strategies based on real-time activity measurements during behavior.

## Graphical abstract

**Figure:**
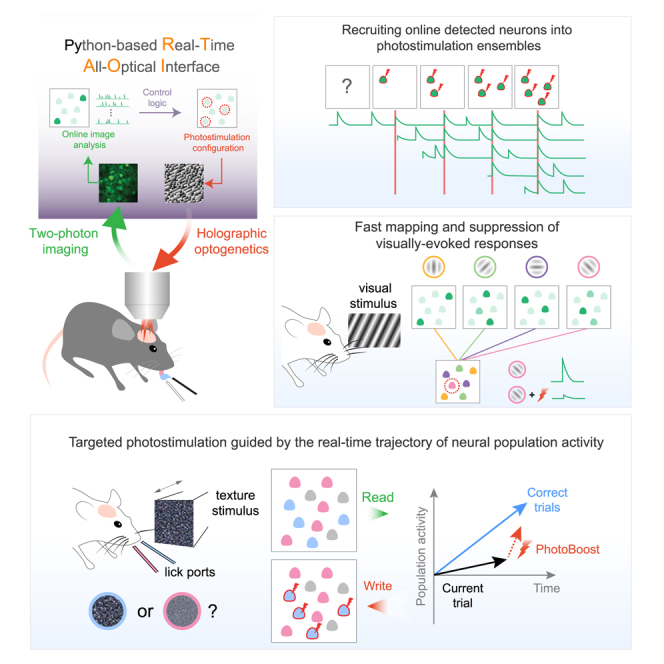

## Teaser

Zhang et al. develop a system that reads activity from individual neurons in real time and uses holographic light patterns to control specific neurons in mice, enabling automated, all-optical experiments to study brain circuits during behavior.

## Introduction

Stimulation approaches have long been used for examining the causal role of neural activity in driving perception and behavior.^1^ Optogenetic techniques allow stimulation to be restricted to genetically defined groups of neurons. More recently, an “all-optical” strategy has been introduced, which combines two-photon calcium imaging and two-photon optogenetics to allow targeting to small ensembles of genetically defined neurons based on their functional signature^2^^,^^3^^,^^4^^,^^5^^,^^6^^,^^7^^,^^8^^,^^9^^,^^10^^,^^11^^,^^12^^,^^13^ Despite the power of this approach, the behavioral consequences of neural activity perturbation—even if it is targeted to functionally defined subpopulations as in the all-optical approach—can be complicated: the effects of perturbations may dissipate over trials^14^ and have opposite signs depending on behavioral states.^7^ Furthermore, there is strong evidence that animals use the information carried by spatiotemporal patterns of activity in specific neural circuits to drive appropriate behavior.^15^^,^^16^^,^^17^ Interventions in the activity of the right neurons at precisely the right time point in the development of these neural dynamics are, therefore, key to testing the causal roles of specific activity patterns. This requires interventions to be guided by the ongoing activity in the neural network during behavior. Neural activity-guided optogenetic stimulation has been used to study memory formation and reinforcement learning,^18^^,^^19^^,^^20^ as well as to interrupt epileptic patterns of activity^21^^,^^22^; however, these experiments have activated large groups of genetically defined neurons without dissecting their functional identities.^23^ We previously introduced a closed-loop all-optical approach that achieved targeted activation of functionally identified neurons or an arbitrarily chosen group of neurons based on the activity of one or a few neighboring neurons.^24^ Recently, holographic optogenetic stimulation of individual neurons has been paired with online behavioral readout in a closed-loop manner,^25^ and online calcium imaging analysis has been adopted to assist targeted photostimulation.^26^^,^^27^^,^^28^ However, online manipulation of functional neuronal ensembles based on the ongoing population dynamics during behavior has not been demonstrated. Being able to achieve such an experimental strategy may reconcile the sometimes conflicting findings of the effects of cortical perturbation on neural circuits and behavioral outputs.^7^^,^^10^^,^^11^^,^^13^

Here, we describe a strategy that enables experiments where circuit manipulation is guided by the real-time readout of neural activity from behaving mice.^29^ We developed an open-source software package, pyRTAOI, which integrates a state-of-the-art calcium imaging analysis suite (CaImAn) to achieve online activity readout from calcium imaging data with accuracy comparable to post hoc analysis.^30^^,^^31^ Together with custom hologram and photostimulation control modules, the toolkit enables flexible control over pre-defined or online-detected neurons. These upgrades from our previous work^24^ not only improved online analysis scale and accuracy but also offer full functionality for efficient execution of complex all-optical behavioral experiments by automating essential steps. Proof-of-principle experiments in two cortical sensory areas demonstrate that neurons can be automatically detected and recruited into a photostimulation ensemble, targeted closed-loop inhibition can be implemented immediately after mapping the orientation preferences of neurons in L2/3 of the primary visual cortex (V1), and targeted activation can be guided by the ongoing activity in a selected population of neurons in L2/3 of the primary somatosensory cortex (S1) while a mouse is performing a tactile discrimination task.

## Results

### A closed-loop all-optical system

**Figure 1:**
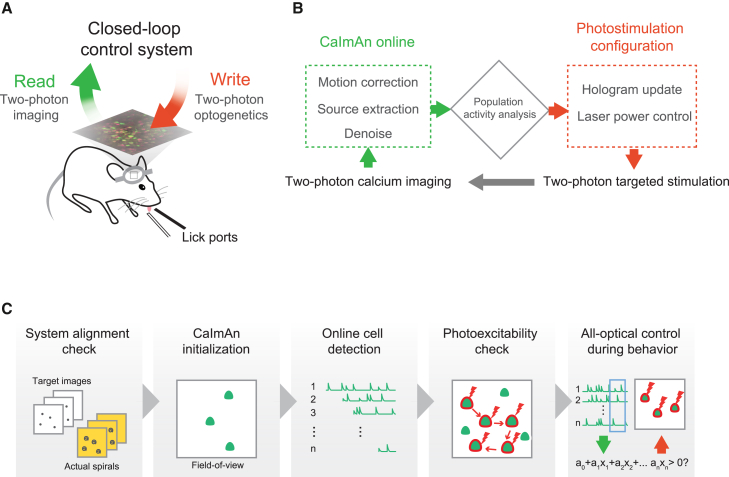
Dynamic closed-loop all-optical control of neural circuits (A) Schematic illustration of the closed-loop all-optical method. (B) The real-time all-optical interface integrates state-of-the-art online calcium analysis algorithms^31^ and a custom holographic photostimulation control module to enable dynamic control over the activity of neural populations with minimal prior knowledge of the underlying field of view. (C) General workflow for the implementation of the closed-loop all-optical method. See also Figure S1 and Table S1.

We developed a closed-loop all-optical system to allow dynamic, targeted photostimulation driven by the real-time activity patterns in a local network involving hundreds of neurons. It consists of two open-source software modules: a Python-based, real-time all-optical interface (pyRTAOI), which communicates with a dual-beam-path two-photon microscope,^9^ performs online image processing using a state-of-the-art calcium image analysis method (CaImAn^31^), and configures photostimulation holograms and laser power via a custom spatial light modulator (SLM) and hologram control software package (Figures 1A, 1B, S1A, and S1B). The system automates and simplifies several essential steps for running complex all-optical experiments (Figure 1C). The general workflow includes the following elements.

#### System alignment check

To test the alignment of imaging and photostimulation pathways prior to an experiment, a series of spot patterns are “burned” in a fluorescence slide using the photostimulation laser and imaged using the imaging laser.^32^ The coordinates of the spots in the pattern are sent to the hologram control software HoloBlink. The phase masks for each pattern are calculated on the fly and displayed on the SLM. The control voltage to an acousto-optic modulator (AOM) is adjusted according to the number of spots to modulate the photostimulation laser power. An overlap of the burnt spots and the target patterns confirms good alignment between the photostimulation and the imaging beam paths, as well as normal system operation (i.e., synchronization and communication between the software and hardware parts of the system).

#### CaImAn initialization

Once the system has been calibrated, two-photon calcium imaging is performed in an experimental animal. A short movie (e.g., 500 frames, ∼17 s) is taken for initializing the CaImAn Online method.^31^ One or a few regions of interest (ROIs) are detected as the “seeds” for the subsequent cell detection session.

#### Online cell detection (optional)

The raw data from the microscope are streamed to CaImAn to extract neuronal activity and define spatial footprints. This session can be completed while the animal is presented with sensory stimuli or while the animal is performing a behavioral task (usually 15–25 min), such that the functional identities of the neurons can be immediately quantified after the session.

#### Photo-excitability check (optional)

The user loads an image containing the centroids of the targets. A series of laser spirals are then targeted at the neurons of interest one by one in a random sequence to test if the photostimulus can evoke activity (measured using calcium imaging). This can be assisted by an opsin expression estimation from the fluorescence molecule tagged with the opsin (see STAR Methods).

#### Closed-loop all-optical control

The user loads a file specifying the indices of neurons, their corresponding weights, and a threshold. The calcium activity of a selected neuron or the weighted sum of the activity of a group of neurons is compared with the threshold at defined time points (e.g., at the time when a stimulus is presented). The threshold-crossing event can be used for triggering photostimulation of pre-defined neurons. Alternatively, photostimulation can also be targeted to newly detected neurons (without running cell detection and photo-excitability check sessions) during the same session, as we will show below.

For optimal performance, we recommend using the faster variants of the GCaMP sensor (GCaMP7f or later variants^33^) because the real-time performance is partly dependent on the speed of the sensor. A GPU (we provide CUDA code for CUDA 8.0 compatible NVIDIA GPUs, e.g., NVDIA GeForce GTX 1080Ti, as used in this paper) is required for converting the raw data from the microscope (Bruker) into images for CaImAn analysis. The online processing—including raw data conversion, motion correction, signal extraction, cell detection, and photostimulation configuration—takes 14.5 ms per frame on average. The processing time is positively and linearly correlated with the number of ROIs being tracked (50 μs per ROI on average) but is able to keep pace with the image acquisition speed (33.3 ms per frame) even when approximately 300 ROIs are tracked online (Figures S1C and S1D).

Below, we present a series of use cases where we implement and validate the closed-loop approach to perform specific manipulations.

### Recruiting online-detected neurons into photostimulation ensembles

The ability to target photostimulation to cells with a particular activity or response pattern is a powerful approach for efficient interrogation of neural circuits. For example, to quickly map the functional connectivity in a network, one may start from a seed neuron that has been detected in the initialization movie and redirect photoactivation to potential follower neurons, such that the local circuitry can be traced progressively.

**Figure 2:**
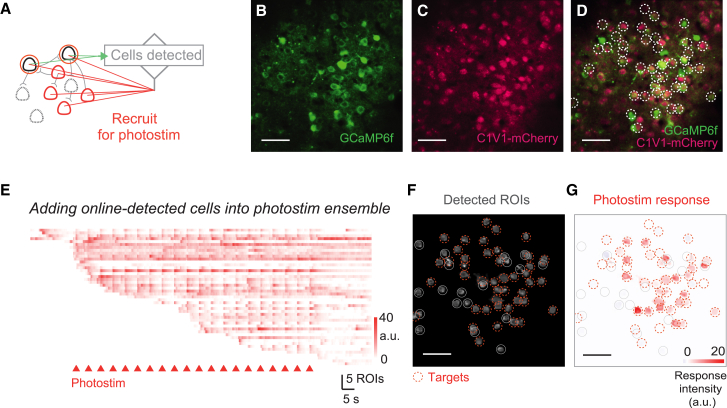
Recruiting online-detected neurons into photostimulation ensembles (A) Experimental strategy for online recruitment of photostimulation targets: putative opsin-positive cells are added into photostimulation ensemble as soon as they are detected. (B–D) A field of view in L2/3 S1 in an awake mouse co-expressing GCaMP6f and C1V1 (scale bar, 50 μm). White circles in (D) mark the online-detected and photostimulated cells. (E) Online-extracted calcium activity in the progressively detected ROIs. Photostimuli were delivered to putative opsin-positive ROIs every 5 s (red triangles, stimulation duration 15 × 10 ms spirals, 6 mW per cell). (F) Spatial footprints of the detected ROIs at the end of the session. (G) Average photostimulation-evoked response. Red circles mark cells that were stimulated at least once; gray circles mark detected ROIs with low mCherry signals that were not targeted.

Here, we demonstrate an experiment in the barrel cortex where cells are detected directly from the live calcium imaging stream and recruited into the photostimulation ensemble in real time (Figure 2). The session started with 4 cells detected in a short movie (∼17 s) for initializing the online calcium analysis pipeline.^31^ During the photostimulation session, the spatial loci of newly detected, opsin-positive cells were immediately sent to a custom photostimulation control module (HoloBlink; see STAR Methods) and were stimulated in the next trial. Thus, active neurons in the FOV were progressively added to the photostimulation ensemble as they were detected (Figure 2E). On average, ∼40 neurons (42.3 ± 10.3, mean ± SD) were detected in the short, 2-min movies, and approximately 40% of them responded to photostimulation (42% ± 9.0%, *n* = 6 FOVs in 3 animals). This result, based on the fast online test, is comparable to that provided by photostimulation-responsiveness mapping routines in which groups of pre-identified opsin-positive cells were randomly activated, which is a far more time-consuming approach.^24^^,^^32^ These results show that the closed-loop approach can automatically target photostimulation to arbitrary cells as they are detected online.

### Fast mapping and suppression of visually evoked responses

Recently developed all-optical methods feature the ability to address sets of neurons based on their functional signature.^4^^,^^5^^,^^7^^,^^8^^,^^9^^,^^10^^,^^11^^,^^12^^,^^13^ However, their functional identities are normally characterized based on baseline sessions recorded hours or days prior to the photostimulation experiments due to the long time typically required for analyzing calcium imaging data. This can be problematic when running behavioral experiments, as the state of the animal and the neural circuit may have changed during the delay. Ideally, a perturbation experiment should start immediately once sufficient information has been collected about the functional properties of the underlying neural circuit.

**Figure 3:**
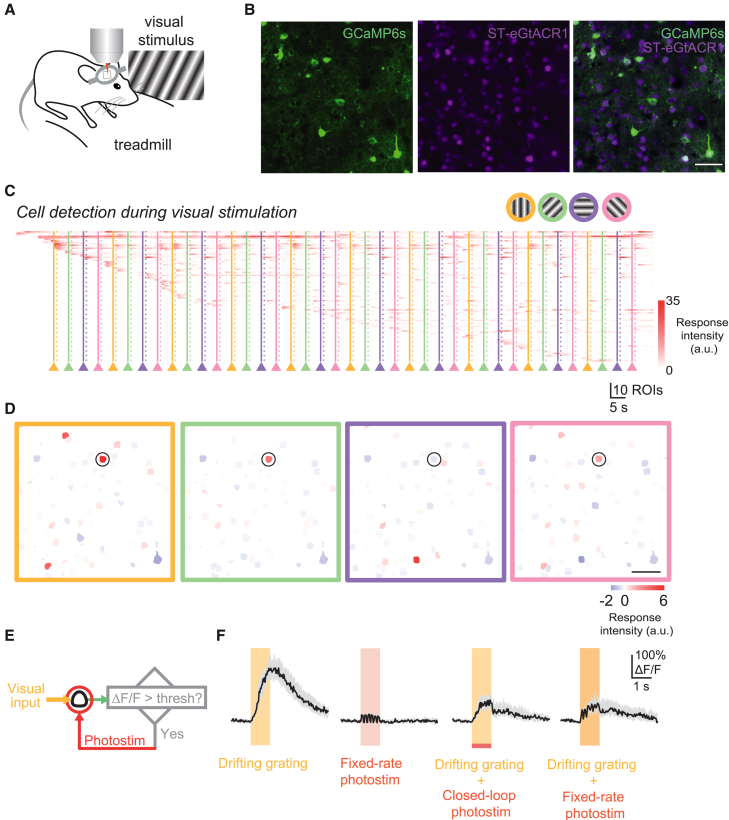
Fast mapping and suppression of visually evoked responses in layer 2/3 V1 (A) Schematic of the experimental setup: a head-fixed mouse on a running wheel is presented with full-screen drifting gratings as a visual stimulus. (B) A field of view in L2/3 V1 co-expressing GCaMP6s and ST-GtACR1 (scale bar, 50 μm). (C) Online-extracted calcium activity in ROIs progressively detected from the FOV as drifting grating stimuli were presented to the mouse once every 5 s. Solid and dotted vertical lines mark onsets and offsets of each visual stimulus (1 s duration), respectively; each color represents a grating stimulus of a particular orientation. (D) Average calcium activity evoked by each visual stimulus. Same color code is used for orientations as in (C). (E) Experimental strategy for closed-loop suppression of visual stimulus-evoked activity. A stimulus-responsive cell (black circle) was selected for the all-optical experiment. (F) Left to right, the activity of the cell in (D) during trials with drifting grating stimuli (vertical grating), fixed-rate photostimulation alone (red area, 6 Hz, 1 s, 10 × 10 ms spirals per stimulation), closed-loop photostimulation during the presentation of the grating stimuli, and fixed-rate photostimulation during grating stimuli (5 s inter-trial interval, 20 trials per trial type). Black trace and shaded area show median and interquartile range, respectively. Note that the traces were contaminated by photostimulation-induced artifacts during the photostimulation window, but the activity level can be estimated from the long tail of the GCaMP6s signal.

Here, we demonstrate an experimental strategy where the orientation tuning properties of neurons in L2/3 V1 are quickly mapped before conducting a closed-loop all-optical inhibition experiment. In the mouse V1, neurons with different visual orientation preferences are spatially intermingled in a “salt-and-pepper” organization.^34^ In order to target photostimulation to neurons with specific orientation tuning, the neuronal responses to differently oriented grating stimuli need to be quantified. A mouse co-expressing the calcium indicator GCaMP6s and the soma-targeted inhibitory opsin ST-eGtACR1^5^ in L2/3 pyramidal neurons in the V1 was presented with a set of drifting gratings of four different orientations (Figures 3A and 3B). Active ROIs were progressively detected during imaging, and their responses to different visual stimuli were immediately quantified at the end of the imaging session (Figures 3C and 3D). One opsin-positive cell (based on the mRuby signal that reflects opsin expression; see STAR Methods) that showed a preferential response to the vertical grating (marked by the black circle in Figure 3D) was selected for an all-optical experiment. To test if the visually evoked activity of the cell can be suppressed on a trial-by-trial basis, targeted optogenetic photoinhibition was applied in either a closed-loop or open-loop fashion. In the closed-loop trials, a short photostimulus was delivered to the cell (10 mW, 100 ms) only when its activity was significantly higher than baseline level, whereas in the open-loop trials, a photostimulus of the same power and duration was delivered at a fixed rate (6 Hz) on top of the visual stimulus (Figure 3F). These two types of photostimulation trials were randomly interleaved with control trials in which only visual stimuli or photostimuli (6 Hz) were presented. Although the photostimulation rate used in the closed-loop trials is significantly lower (4.2 ± 0.39 Hz, *p* < 0.0001, 80 trials for either condition, 4 cells), the average activity immediately after the photostimulation window in closed-loop trials is similar to that in open-loop trials (*p* = 0.64, paired t test, 4 cells in 2 animals). This means that the closed-loop photoinhibition, where photostimuli were titrated based on the activity of the cell itself, functioned as well as the open-loop suppression, where stronger photostimuli were delivered at a fixed rate. These results demonstrate that by harnessing the efficiency gained by the online analysis, the closed-loop toolkit allows activity-guided optogenetic intervention immediately after circuit characterization.

### Targeted photostimulation guided by the real-time trajectory of neural population activity during behavior

Since neural activity in the cortex is highly dynamic and variable, the impact of injecting a particular activity pattern into a cortical network can change depending on the state of the network.^35^^,^^36^ A powerful way to probe the behavioral relevance of a particular activity pattern is to activate or disrupt it during specific network states. Specifically, in trials when activity related to the correct choice is absent and an incorrect choice or a miss trial is imminent, one could use closed-loop photostimulation to inject specific activity patterns into the network to see whether it is possible to rescue the behavior or to improve behavioral performance.

**Figure 4:**
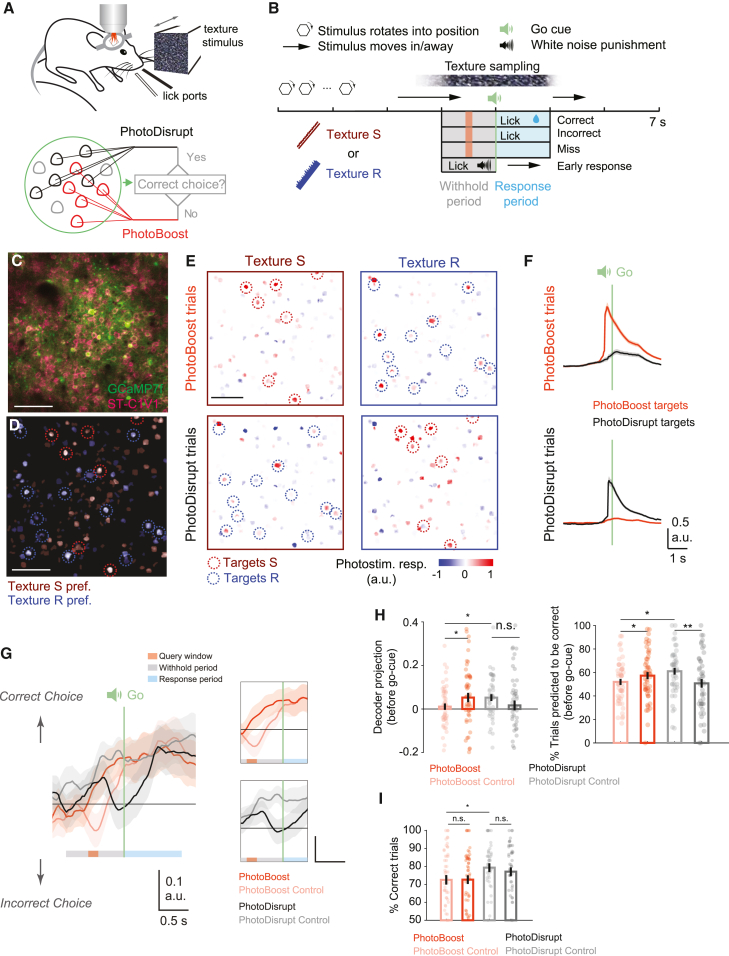
Closed-loop photostimulation guided by neural population activity in layer 2/3 barrel cortex of mice solving a texture discrimination task (A) Schematic of the experimental setup, featuring a head-fixed mouse trained to lick one of the two lickports for rewards based on which texture stimulus is presented. Photostimulation is delivered depending on the upcoming choice predicted from the underlaying circuit, targeting either the neuronal ensemble that encodes the presented texture to boost the circuit representation or the ensemble that encodes the other texture to disrupt the ongoing activity. (B) Trial structure of the two-choice texture discrimination task. Orange bar: photostimulation query window (5 frames, 600–433 ms before go cue). (C) An example FOV in layer 2/3 barrel cortex co-expressing calcium indicator (GCaMP7f) and soma-targeted opsin (ST-C1V1). (D) Subsets of neurons preferably responsive to the rough and smooth textures (Stim R and Stim S). (E) Photostimulation response in the example FOV when either of the two target ensembles was stimulated during the task. (C–E) Scale bars, 50 μm. (F) Trajectories of the calcium signal in the stimulated neurons in the PhotoBoost and PhotoDisrupt trials. Top: PhotoBoost targets; bottom: PhotoDisrupt targets. (G) Population activity projected onto the choice decoder axis. (H) Left: choice decoder projections averaged from the beginning of the query window to the onset of go cue. Right: choice predictions based on the average decoder projections. (I) Behavioral outcomes in different trial types. *n* = 50 conditions for PhotoBoost/control and 47 conditions for PhotoDisrupt/control, 7 mice. Wilcoxon signed-rank test was used to compare condition and control trials; Wilcoxon rank-sum test was used to compare PhotoBoost and PhotoDisrupt. ∗*p* < 0.05. See also Figure S2-4. Shaded areas in (F) and (G) and error bars in (H) and (I) are SE.

Here, we demonstrate a proof-of-principle experiment where targeted photostimulation is guided by the population activity of neurons in L2/3 barrel cortex during a texture-based sensory decision-making task. Mice were trained to perform a two-choice texture discrimination task while head-fixed and allowed to run freely on a running wheel. One of two texture stimuli (smooth or rough sandpaper, Stim S or Stim R) was presented in each trial.^17^ Mice reported the identity of the stimulus by licking one of two lickports associated with each stimulus (Figures 4A and 4B). At the start of each trial, one texture was moved into contact with the whiskers to allow free sampling of the sandpaper. Approximately 2 s into the sampling period, an auditory “go cue” signaled the start of the response window, within which licking the lickport associated with the texture triggered a sugar water reward. During texture sampling, subsets of neurons were preferentially responsive to either the smooth or rough sandpaper when the mice made the correct choices (Figures 4C and 4D). A linear decoder was built based on the activity recorded from these texture-responsive neurons in a baseline session to predict if the mouse would make a correct choice in a given trial. In the closed-loop session, the real-time activity patterns extracted from the calcium signals were projected onto the choice decoders in a short query window (5 frames, 600–433 ms) before the go cue. In half of the trials where the mouse was predicted to make a wrong choice during the query window, a brief photostimulus was triggered to activate the target ensemble whose trial preference aligns with the presented stimulus type and correct choice (PhotoBoost). The other half of the trials where a wrong choice was predicted were used as controls for the PhotoBoost condition (PhotoBoost control). Alternatively, in 1/3 of the trials when the decoder predicts a correct choice, the target ensemble that prefers the opposite trial type was activated (PhotoDisrupt) at the end of the query window, and the other 2/3 of the predicted correct trials were used as controls for the PhotoDisrupt condition (PhotoDisrupt control; Figure 4E). This activity-based, functionally targeted photostimulation biased the activity trajectories in different directions depending on the ongoing population activity during the task (Figures 4G and 4H), although we did not observe a behavioral change induced by photostimulation. This experiment demonstrates that our all-optical closed-loop strategy can detect and test the behavioral relevance of activity patterns “on the fly” during perceptual decision-making.

## Discussion

We describe a strategy based on a set of integrated software and hardware tools for dynamic control of behaviorally relevant activity of individual neurons in the mouse cortex. Integrating an online calcium imaging analysis strategy with custom modules, the software allows easy implementation of various types of all-optical experiments. We have demonstrated three types of proof-of-principle experiments using this strategy. First, active neurons were automatically detected and recruited into the photostimulation ensemble. This online recruitment of photostimulation targets may, in the future, be used to explore circuit connectivity or to suppress spontaneous epileptic activity in the network.^21^^,^^22^ Second, the orientation tuning properties of neurons in the visual cortex were characterized using online-processed imaging data, which was immediately followed by selective closed-loop inhibition of visually evoked activity. This demonstrates that the toolkit provides an efficient way of conducting all-optical experiments with a seamless transition from circuit characterization to circuit interrogation. Third, functional ensemble-specific activation was guided by the population activity in the barrel cortex of a mouse solving a tactile discrimination task. This activity-guided photostimulation is the first demonstration of a dream experiment where targeted circuit perturbation is directed by the ongoing neural activity as it evolves during behavior.

Compared to other closed-loop neural control strategies,^21^^,^^22^^,^^37^^,^^38^ the two-photon all-optical approach has the advantage that it can be readily targeted to genetically and functionally defined cell types and provides flexible control over the number and spatial distribution of both the neurons for readout and those for targeted stimulation. The toolkit we developed here also provides substantial improvements to our previously described closed-loop all-optical system^24^ (Table S1). First, the online activity readout is not limited to one or a few cells but has been extended to hundreds of neurons across the entire FOV with an accuracy comparable to that of offline analysis.^31^ Second, photostimulation is no longer restricted to pre-defined targets but can be redirected to arbitrary neurons, even onto neurons that were not detected before the online experiment. In a proof-of-principle experiment, we targeted photostimulation only to the opsin-positive cells by applying a binary “opsin mask.” Other masks reflecting the functional or genetic identity of the neurons can also be used to restrict the recruitment to specific neuronal populations. Third, it minimizes a time-consuming step in conventional all-optical experiments, which is to identify functional ensembles from a baseline imaging session and test if they can be activated optogenetically. A recent study also adopted CaImAn Online to accelerate this process.^26^ The software we developed here not only processes raw imaging data on the fly but also automates the procedure for photo-excitability screening. Finally, the open-source software in Python is easy to implement with other imaging systems and analysis toolboxes. It also provides a user-friendly interface to flexibly configure various experimental protocols. Compared to recently published closed-loop all-optical toolboxes,^27^^,^^28^ our software takes full advantage of state-of-the-art imaging analysis methods, with functions optimized to track and interfere with neuronal activity during goal-directed behavior (Table S1). Here, we demonstrate its usefulness in studying the superficial layers in the cortex, but this method can, in principle, be applied to any brain area accessible to two-photon microscopy, including deep brain regions, for example, by implanting a gradient index (GRIN) lens over the ROI.^12^ Closed-loop experiments based on optical methods have been a staple of brain-computer interfaces.^38^^,^^39^ The method proposed here could ultimately be adopted to assist neuroprosthetic learning by boosting the neural response with targeted stimulation. Together, the results of this approach open up many opportunities for all-optical circuit interrogation.

### Limitations of the study

First, the temporal resolution of the current system is limited primarily by the relatively slow activity readout from calcium sensors. Even with the use of the latest genetically encoded calcium sensors, the fastest circuit dynamics, on the millisecond timescale, are challenging to capture using calcium indicators^40^ (the latest GCaMP variants exhibit a rise time of tens of milliseconds in response to a single spike^33^^,^^41^^,^^42^). The use of genetically encoded voltage sensors, enabling voltage deflections associated with single spikes to be detected directly,^43^^,^^44^^,^^45^ can, in principle, speed up the closed-loop timescale by up to two orders of magnitude. However, further development is needed to investigate efficient means for online image analysis and high-speed imaging strategies that can follow the fast voltage sensor kinetics across large numbers of cells *in vivo*.^46^^,^^47^^,^^48^^,^^49^^,^^50^^,^^51^

Second, the spatial scale of the current approach is limited to a 2D plane of ∼400 × 400 μm. On the imaging side, the maximum number of neurons for online activity tracking is ∼600 in order to maintain a 30 Hz image acquisition rate. On the photostimulation side, the maximum number of target neurons is currently under 50, limited by the hologram size and the co-expression of GCaMP and opsin. Future experiments may use viral constructs that contain both GCaMP and opsin genes^11^ or transgenic mice^26^ to ensure optimal co-expression of the two proteins. The closed-loop method can, in principle, be extended to three-photon microscopy to reach deeper brain regions.^52^ Furthermore, the use of advanced microscopy designs, such as rapid random-access scanning^53^^,^^54^^,^^55^ and parallel processing units,^23^ will further extend the addressable FOV to three dimensions.

Third, the fluorescence artifact induced by the photostimulation laser prevents a reliable calcium activity readout during the stimulation. Since prolonged photostimulation light is often required by inhibitory opsins, the artifact limits the number of cells that can be simultaneously suppressed. Here, we demonstrated examples of single-cell photoinhibition, with future work required to test the efficiency of closed-loop photoinhibition with a larger number of samples, to extend the method to inhibiting multiple cells, and to deliver photostimulation without the knowledge of the timing of sensory stimuli. Artifact mitigation strategies, such as laser pulse timing gates^5^ or computational subtraction, could be adopted to allow measurement during photostimulation, such that the photostimulation laser can remain on while adjusting the holographic pattern to achieve control over all cells in an ensemble.

Finally, in a proof-of-principle experiment, we attempted to detect and correct erroneous decision signals in the barrel cortex of mice, which are sparse compared to those in downstream areas and are difficult to capture with a simple linear model. Future work is needed to build an improved decoder for online prediction of choice from high-dimensional neuronal activity. Other experimental protocols may also be implemented, for example, PhotoBoost during trials when the animal is predicted to make a correct movement to test the circuit activity and behavioral outcomes. Nevertheless, the approach we have described promises to be a powerful tool for investigating decision-making processes in higher-order brain regions such as the posterior parietal cortex, the secondary motor cortex, and the anterior lateral motor cortex, where recent work has shown clear divergence in the activity trajectories preceding alternative choices in two-photon calcium imaging experiments.^4^^,^^56^^,^^57^^,^^58^^,^^59^^,^^60^ Such a strategy could form the basis for online choice decoding and evaluation of the circuit impact of optogenetic perturbations.

## Resource availability

### Lead contact

Requests for further information and resources should be directed to and will be fulfilled by the lead contact, Michael Häusser (m.hausser@ucl.ac.uk).

### Materials availability

This study did not generate new unique reagents.

### Data and code availability

• All data reported in this paper will be shared by the lead contact upon request.
• The software code together with detailed instructions can be found at on Zenodo (https://doi.org/10.5281/zenodo.16424873) and GitHub (https://github.com/alloptical/pyRTAOI).
• Any additional information required to reanalyze the data reported in this paper is available from the lead contact upon request.

## Acknowledgments

We thank Hillel Adesnik (UC Berkeley) for providing the ST-eGtACR1 virus; Selmaan Chettih and Christopher Harvey (Harvard Medical School) for sharing the soma-targeted C1V1 virus; Adam Packer for advice on the microscope setup and for mentoring Z.Z. in the early stages of the project; and Isaac Bianco, Arnd Roth, and Alex Prodan for discussions and feedback. We are grateful to Soyon Chun and Caroline Reuter for technical support. This work was supported by grants from the 10.13039/100010269Wellcome Trust (PRF 201225 and 224688), the ERC (AdG 695709), the MRC (MR/T022922/1), and the 10.13039/501100000268BBSRC (BB/N009835/1).

## Author contributions

Z.Z. and M.H. conceived the project; Z.Z. and P.D. developed the closed-loop software; Z.Z. performed the experiments and analyzed data; Z.Z., L.E.R., and O.M.G. did the surgeries; L.E.R. developed the visual stimuli and behavior control software; R.R., C.B., and Z.Z. trained the animals; C.B. built the texture stimuli setup and mentored the texture experiments; D.R.S. and M.H. supervised Z.Z.; and Z.Z., P.D., and M.H. wrote the manuscript.

## Declaration of interests

The authors declare no competing interests.

## STAR★Methods

### Key resources table

**Table:** 

REAGENT or RESOURCE	SOURCE	IDENTIFIER
**Bacterial and virus strains**		

AAVdj-CaMKIIa-C1V1(E162T)-TS-P2A-mCherry-WPRE	Stanford Vector Core	GVVC-AAV-46
AAV9-FLEXED-C1V1-Kv2.1-mRuby2	Christopher Harvey Lab	N/A
pAAV-CAG-DIO-NLS-mRuby3-IRES-eGtACR1-ST	Hillel Adesnik Lab	N/A
AAV1-*syn*-jGCaMP7f-WPRE	Addgene	104488
AAV-CAMKII-0.4Cre-SV40	Addgene	105558

**Experimental models: Organisms/strains**		

Wild-type mice, C57BL/6J	Charles River	Strain code: 027

**Software and algorithms**		

pyRTAOI: custom software for data processing	This paper	GitHub link: https://github.com/alloptical/pyRTAOI Zenodo link: https://doi.org/10.5281/zenodo.16424873
Suite2P	Pachitariu et al., 2017^61^	https://github.com/MouseLand/suite2p
CaImAn	Giovannucci et al., 2019^31^	https://github.com/flatironinstitute/CaImAn
PrairieView	Bruker	https://bruker-control.readthedocs.io/en/latest/
Blink Overdrive Plus	Meadowlark Optics	https://www.meadowlark.com/spatial-light-modulators/
GenerateHologramCUDA	HotLab	https://github.com/MartinPersson/HOTlab/tree/master/GenerateHologramCUDA_dll
PyBehaviour: behavioral control software	Russell et al., 2022	https://github.com/llerussell/PyBehaviour
PackIO	Watson et al., 2016^62^	PackIO: https://apacker83.github.io/PackIO/
Python	Python.org	RRID: SCR_008394
MATLAB	Mathworks	RRID: SCR_001622
Spyder	Anaconda.org	https://anaconda.org/anaconda/spyder
Visual studio	Microsoft	https://visualstudio.microsoft.com/
CUDA	NVIDIA	https://developer.nvidia.com/cuda-toolkit
Adobe Illustrator	Adobe	RRID: SCR_010279

### Experimental model and study participant details

All experimental procedures were carried out under license from the UK Home Office in accordance with the UK Animals (Scientific Procedures) Act (1986). Adult mice (8–12 weeks old, both male and female, C57BL/6J wild type, Charles River Laboratories) were used for experiments. Mice used for textile behavioral experiments were singly housed to prevent cage-mates pulling their whiskers and were transferred to a daylight reversal chamber and water restricted (at the earliest one-week post-surgery) to increase motivation for behavioral tasks.

### Method details

#### Animal preparation

At the beginning of surgeries, mice were given a subcutaneous injection of 0.3 mg/mL buprenorphine hydrochloride (Vetergesic) and anesthetized with isoflurane (5% for induction, 1.5–2.5% for maintenance). Lidocaine was applied to the skin for local anesthesia before removing the scalp above the dorsal surface of the skull. A metal headplate with a 7 mm diameter circular imaging well was fixed to the skull centered over V1 (2.5 mm lateral and 0.5 mm anterior from lambda) or S1 (1.6 mm posterior and 3.5 mm lateral of bregma) with dental acrylic (Super-Bond C&B, Sun-Medical). A 4 mm diameter craniotomy was drilled inside the imaging well. 1 μL of a mixture of AAV1-*syn*-jGCaMP7f-WPRE (dilution 1:15), AAV9-FLEXED-C1V1-Kv2.1-mRuby2 (gift from Chris Harvey,^10^ dilution 1:30), and AAV-CAMKII-0.4Cre-SV40 (1:15 dilution) or AAV1-hSyn-GCaMP6s-WPRE-SV40, AAV9-CAG-DIO-NLS-mRuby3-IRES-eGtACR1-ST(gift from Hillel Adesnik^5^) and AAV-CAMKII-0.4Cre-SV40 (1:10 dilution) virus was injected into layer 2/3 (∼300 μm deep) at 0.1 μL/min. The dura was removed after virus injection. A cranial window (4 mm diameter circular glass coverslip) was press-fit into the craniotomy and sealed using cyanoacrylate (Vetbond) before fixing with dental cement. At the earliest one week after the surgery mice were transferred to a daylight reversal chamber and water restricted to increase motivation for behavioral tasks.

#### Two-photon imaging and photostimulation

Experiments were carried out using a commercial all-optical microscope (Bruker) as described previously.^24^ A 25×/0.95-NA objective (Leica) was used for experiments in Figures 1 and 2 (268 × 268 μm FOV), and a 16×/0.8-NA objective (Nikon) was used for the texture behavioral experiments for imaging across a larger FOV (416× 416 μm). The power control module for the photostimulation laser, initially a Pockels cell, was replaced with an AOM (MCQ80-A2-L1064-Z32, AA Opto-Electronic) for faster modulation. All photostimulation experiments were performed using a Satsuma laser (Satsuma HP2, Amplitude; pulse repetition rate, 2 MHz). A reflective multilevel phase SLM was used to display holograms (OverDrive Plus SLM, Meadowlark Optics/Boulder Nonlinear Systems; 7.68 × 7.68 mm active area, 512 × 512 pixels, optimized for 1064 nm).

#### The real-time all-optical software toolkit

We developed a Python-based, real-time all-optical interface (pyRTAOI, written in Python 3.6 with PyQt5, developed in Spyder 3.2.4) that integrates the toolbox CaImAn^31^ for online calcium imaging analysis and can perform different types of targeted photostimulation based on user configurations. It also provides users with access to real-time motion-corrected and denoised imaging frames, calcium activity traces recorded from more than a hundred regions-of-interest (ROIs), manual curation and selection of cells for readout and stimulation, configuration of photostimulation and sensory stimulation protocols (e.g., laser power, frequency, amplitude, duration, etc.), automatic photostimulation and imaging laser alignment check, automated detection of opsin-positive cells and direct control of relevant parameters settings in CaImAn and the Prairie View microscope system (Figure S1). Raw data from the microscope system was converted into frames of 512 × 512 pixels accelerated by a GPU (CUDA8.0, NVDIA GeForce GTX 1080Ti) and downsampled to 256 × 256 pixels before passing to CaImAn for calcium image processing. The image processing works in parallel with image acquisition on a separate thread, which includes motion correction and neuronal source extraction to generate denoised calcium activity traces for neurons in the FOV. The processing time for each frame is checked and displayed. pyRTAOI will stop and save out results files automatically when no more frames are available in the PrairieView data buffer. The user can abort a session at any time by stopping acquisition in PrairieView. Users can configure control logics to use the activity from selected neurons (e.g., a linear combination of their instant calcium signals) to determine the photostimulation targets and strength (i.e., stimulation timing, duration and the laser power), which is passed to the custom SLM control software.

To enable flexible targeting for optogenetic photostimulation, we developed an SLM control interface, ‘HoloBlink’ (written in C++ with Qt 5.9, Microsoft Visual Studio 2013, Figure S1). Upon receiving a command which declares the coordinates of the photostimulation targets, the software transforms the coordinates from the imaging space to photostimulation space, weights the points by the distribution of light intensity over the FOV, calculates the 2D hologram using the weighted Gerchberg-Saxton algorithm with a GPU (CUDA8.0, NVIDIA GeForce GTX 1080Ti, typically <15 ms when <20 targets are required)^63^ and then displays it on the SLM using the API from Meadowlark Optics. The time budget for online processing is summarized in Figures S1C and S1D. The photostimulation laser power was adjusted according to the number of targets by changing the voltage applied to the AOM via an analog output device (NI-DAQmx, PCI-6713, National Instruments). The power control is synchronized with spiral scanning by the microscope system by a TTL pulse trigger. The software code together with detailed instructions can be found at the following GitHub link: https://github.com/alloptical/pyRTAOI. Below are the key technical requirements for the closed-loop system.

• A dual beam path two-photon microscope for *in vivo* imaging and photostimulation (e.g., from Bruker, Thorlabs, custom build, etc.).
• Microscope control software that gives raw data access (e.g., PrairieView for Bruker systems)
• An SLM with high diffraction efficiency and high speed (e.g., LCoS SLMs from Meadowlark Optics)
• Graphics card for accelerating raw data conversion and computation of phase masks (e.g., GPUs from NVIDIA)
• Data acquisition cards (e.g., National Instruments) and synchronization software (e.g., PackIO: http://www.packio.org)
• AOM for controlling photostimulation laser power
• Python (3.6) with PyQt5
• MATLAB (2018b or later)
• Microsoft Visual Studio with Qt (vs. 2013 with Qt 5.9 or later)

For a comprehensive list of hardware for all-optical experiments, please refer to Russell et al., 2022.^32^ Example use cases for closed-loop all-optical interrogation include.

• Efficient cell-by-cell calibration of photostimulation intensity in all-optical experiments
• Optical “clamping” of spike rates to probe rate coding in neural circuits
• Precisely timed intervention in neural circuits based on evolution of activity patterns
• Boosting or mimicking sensory-evoked activity patterns (e.g., in sensory prostheses)
• Optical brain-machine interfaces.
• Opsin expression estimation

##### Opsin expression estimation

The opsin expression level was estimated from two-photon images of the red fluorescent proteins co-expressed with the opsins: mCherry for the C1V1 virus (AAVdj-CaMKIIa-C1V1(E162T)-TS-P2A-mCherry-WPRE) or mRuby for the ST-eGtACR1 virus (pAAV-CAG-DIO-NLS-mRuby3-IRES-eGtACR1-ST). The image of the red fluorophores (imaged at 765 nm) was binarized using a built-in CaImAn function (extract_binary_masks_from_structural_channel) to obtain an ‘opsin mask’ using an adaptive thresholding method where the binarization threshold for each pixel was calculated as the weighted sum of its neighborhood pixels (cross-correlation with a Gaussian window with standard deviation equals to the expected radius of cell or nuclear). The opsin mask is then compared to the ROI shapes detected from the calcium movie. ROIs are defined from the calcium signal, with most of the pixels (>50%) overlapping with the opsin mask were identified as opsin-positive.

#### Calcium image processing

Extracting neuronal calcium activity from the raw fluorescence data comprises three steps: (1) data conversion that organizes raw data into movies; (2) motion correction that registers the frames within the movie to compensate for spatial shifts of the FOV resulting from brain motion; (3) neuronal signal extraction that detects neurons from the background and separates their calcium fluorescence signal from the neuropil signal. For online processing, raw data streaming from the microscope system, PrairieView (Bruker) was organized into frames of 512 × 512 pixels accelerated by a GPU (NVDIA GeForce GTX 1080Ti). To boost processing speed and reduce noise, frames were downsampled to 256 × 256 pixels. The frame was then passed onto CaImAn for motion correction and neuronal signal extraction. For optimized online performance, CaImAn should be initialized using a short (∼20 s) recoding or a structural mask such as a binary opsin mask. At this step, the initial set of candidate target cells is detected based on their functional or spatial signature. During the online stage, any newly active cells are subsequently detected by the OnACID algorithm^30^ and added into the available cell pool.

Two state-of-the-art calcium imaging analysis packages were used: Suite2P^61^ and CaImAn. Suite2P can detect more cells in some applications than CaImAn, whereas CaImAn provides fast, online implementation of denoising simultaneously with signal extraction (see Pachitariu et al., 2016 and Giovannucci et al., 2019 for detailed comparisons). Therefore, we used Suite2P for post-hoc, offline analysis to obtain more information about the FOV, and CaImAn for fast online analysis. On the day of the experiment, photostimulation targets were selected from neurons detected by CaImAn from the live imaging stream before the recording sessions. For post-hoc analysis, all imaging sessions recorded on the same day were concatenated for Suite2P analysis, and the cells with spatial footprints more than 30% overlapping with a 40-pixel (32.5 μm) diameter circular mask centered at the centroids of the photostimulation spirals are taken as targeted cells. In the texture task sessions, the number of cells detected online and offline was comparable (Figures S3A and S3B). Post-hoc analysis confirmed the texture preference and selective activation of the online-selected target cells (Figures S3C and S3D; S4).

#### Neuronal activity analysis

Receiver operating characteristic (ROC) analysis was used to quantify the trial-wise response of individual neurons to photostimuli or sensory stimuli.^64^ In each trial, the average calcium activity level during the withhold window was taken as the neuronal response to the presented stimulus. Trials in which the animal licked during the withhold window (the ‘early lick’ trials) were excluded from imaging analysis to minimize contamination from locomotion-related signals. For each neuron, ROC curves were generated by comparing the true positive and false positive rate of a binary classifier that uses different threshold to discriminate the neuronal response in two trial types. To avoid possible bias from imbalanced sample size, the maximum area-under-the-curve (AUC) achieved by the optimal threshold was normalized by those computed from shuffled data (generated by shuffle the trial labels, 500 repeats). For quantifying texture selectivity, calcium activity in correct trials were used to compute the normalized AUC values and neurons with texture selectivity larger than 1.5 were taken as texture preferring neurons. Neurons that showed significantly different activity levels during the withhold window compared to baseline (1 s before the trial starts) in correct trials were used to estimate the network activity (referred to as ‘touch neurons’). Calcium activity traces of each neuron during the four types of trials (correct S, correct R, incorrect S, incorrect R) were concatenated in time into a single activity vector. Activity vectors of all touch neurons form a matrix with each column corresponding to one neuron. Demixed principal component analysis (dPCA)^65^ was used to reduce the dimensionality of the matrix to five principal components separating trial-type related components from time-dependent components. A logit model was then fit to the dPCs averaged during the withhold window in correct S or R and incorrect S or R trials to obtain two trial outcome decoders (‘mnrfit’ function in MATLAB2018b with the statistics and machine learning toolbox) for online choice prediction for S or R trials, respectively. The coefficients of the decoders (output ‘B’ of the ‘mnrfit’ function) were multiplied with the weights from dPCA (output ‘W’ of the ‘dpca’ function) to get a vector containing the weight of each touch cell. The touch cell activity vector was multiplied with the weight vector in each frame during the query window for choice prediction (Figure S2). On average per texture type per session, 45.9 ± 3.0 (mean ± s.e.) trials were detected as incorrect, 56.7 ± 0.01 of which were set to PhotoBoost and the rest set to No stimulation; 39.0 ± 2.5 trials were detected as correct, 54.0 ± 0.02 of which were set to PhotoDisrupt and the rest as control. To test if the lack of behavioral effect of the photostimulation was due to the poor online decoder performance, or the photostimulation itself, we constructed an offline decoder based on the neural activity preceding the photostimulation (same as online decoder). Then, the trials were divided into six trial types (1. Predicted to be S + photostimulated targets S (PredictS PhotoS), 2. Predicted to be S + photostimulated targets R (PredictS PhotoR), 3. Predicted to be S + no photostimulation (PredictS NoPhoto), 4. Predicted to be R + photostimulated targets S (PredictR PhotoS), 5. Predicted to be R + photostimulated targets R (PredictR PhotoR), 6. Predicted to be R + no photostimulation (Predict R NoPhoto)). There is no clear behavioral effect across the six trial types, which suggests that the lack of effect of photostimulation is likely due to the choice of targets, the number of target neurons or the timing of the stimulation, rather than representing a limitation of the method itself.

#### Photostimulation target selection

Calcium traces extracted online from the two-photon imaging stream by CaImAn were used for quick analysis on the day of experiments. To identify behaviorally relevant neural populations in the texture discrimination task, cells were first detected from the live imaging stream while the animal started performing the task (normally ∼150–300 ROIs were detected in 15–25 min). Cell detection was then disabled, and imaging and behavioral recording started after the animal made consecutive correct transitions between the two lickports. First, a baseline imaging session consisting of 150 trials was used for mapping the functional identity of cells in the FOV and for building choice decoders. Next, to test which trial-coding neurons can be activated optogenetically, a short photostimulation pulse (9 × 10 ms spiral stimuli delivered at 100 Hz, 7–8 mW per cell) was delivered to each texture-selective cell in a random sequence (0.5 s inter-stimulus interval, 10–15 repeats per cell). This photo-excitability evaluation process is automated in pyRTAOI. Trial-coding neurons that show significant calcium responses to photostimulation (z-scored photostimulation response >1) were grouped by trial preference into two functional target ensembles.

#### Visual stimulation

Visual stimuli were generated using custom software. To map orientation preference, full screen drifting gratings (4 directions, 0° to 135° in 45° increments) with a spatial frequency of 0.04 cycles/° and a temporal frequency of 2 Hz^66^) were presented on a monitor (24″ Dell monitor, ∼15 cm from the animal’s left eye) in a sequence with a duration of 1 s, interleaved by 5 s of full screen, mean luminance gray. For the closed-loop suppression experiment, the preferred grating stimulus was presented in the same manner.

#### Barrel mapping

Wide-field imaging was performed to locate the barrel centers a minimum of 10 days after surgery. Before imaging, all whiskers except C1-3, B1-3, and D1-3 were trimmed to less than 5 mm long while the animals were under isoflurane anesthesia (0.5–1%) to minimize task-relevant activity outside of the photostimulatable area. After the animals recovered from anesthesia, imaging was performed while the animals were head-fixed under the objective and were free to run on a treadmill. Excitation light from a 470 nm LED (M470L3, Thorlabs) passed through an aspheric condenser lens (ACL2520U-DG15, Thorlabs), a filter (ET470/40, Chroma) and was reflected into the back aperture of the objective (4x Nikon Plan Fluorite Imaging Objective, 0.13 NA, 17.2 mm WD) by a 495 nm long-pass dichroic filter (FF495-Di03-25x36, Semrock) to reach the cranial window. Emitted light passed through the same 495 nm long-pass dichroic filter, a 749 nm short-pass dichroic filter (this filter is for the two-photon configuration in the same setup, FF749SDi01-25 × 36 × 3, Semrock) and a bandpass filter (HQ525/50, Chroma) before reaching the camera. Images were acquired at 10 Hz with a camera (ORCA-Flash 4.0 V3, Hamamatsu). In each imaging session, a single whisker was threaded into a glass capillary tube glued to a one-dimensional piezoelectric actuator (PL127.11, Physik Instrumente) and was subject to sinusoidal deflections (10 Hz for 1 s) every 10 s. The centers of the barrel columns were estimated from the stimulus-triggered activity in the fields-of-view by taking the average of the frames aligned to the onsets of whisker stimuli, normalized to the frames before the stimulus onsets.

### Quantification and statistical analysis

Statistical tests are described in figure legends. Scatterplots show least-squares lines, and Pearson’s correlation coefficients were calculated using the MATLAB function “corrcoef”. All data are reported as the mean and standard error of the mean (SEM) unless otherwise stated; n.s., not significant; ∗ *p* < 0.05; ∗∗ *p* < 0.01; ∗∗∗ *p* < 0.001. Details of statistical tests and sample sizes can be found in the results text and in figure legends. No statistical methods were used to predetermine sample size, and we did not use methods to test for normality of sample distributions. Experimenters were not blind to experimental conditions.

## Supplemental information

Document S1. Figures S1–S4 and Table S1

Document S2. Article plus supplemental information
