# Supplementary material for: A real-time all-optical interface for dynamic perturbation of neural activity during behavior
Source: Cell Rep Methods. 2025 Sep 18;5(10):101180. doi: 10.1016/j.crmeth.2025.101180 (PMC12570328; doi:10.1016/j.crmeth.2025.101180)
Supplement: Document S1. Figures S1–S4 and Table S1 [file mmc1.pdf]

Cell Reports Methods, Volume *5*

Supplemental information

A real-time all-optical interface for dynamic perturbation of neural activity during behavior

Zihui Zhang, Patrycja Dzialecka, Lloyd E. Russell, Riccardo Ratto, Christina Buetfering, Oliver M. Gauld, David R. Selviah, and Michael Häusser

A

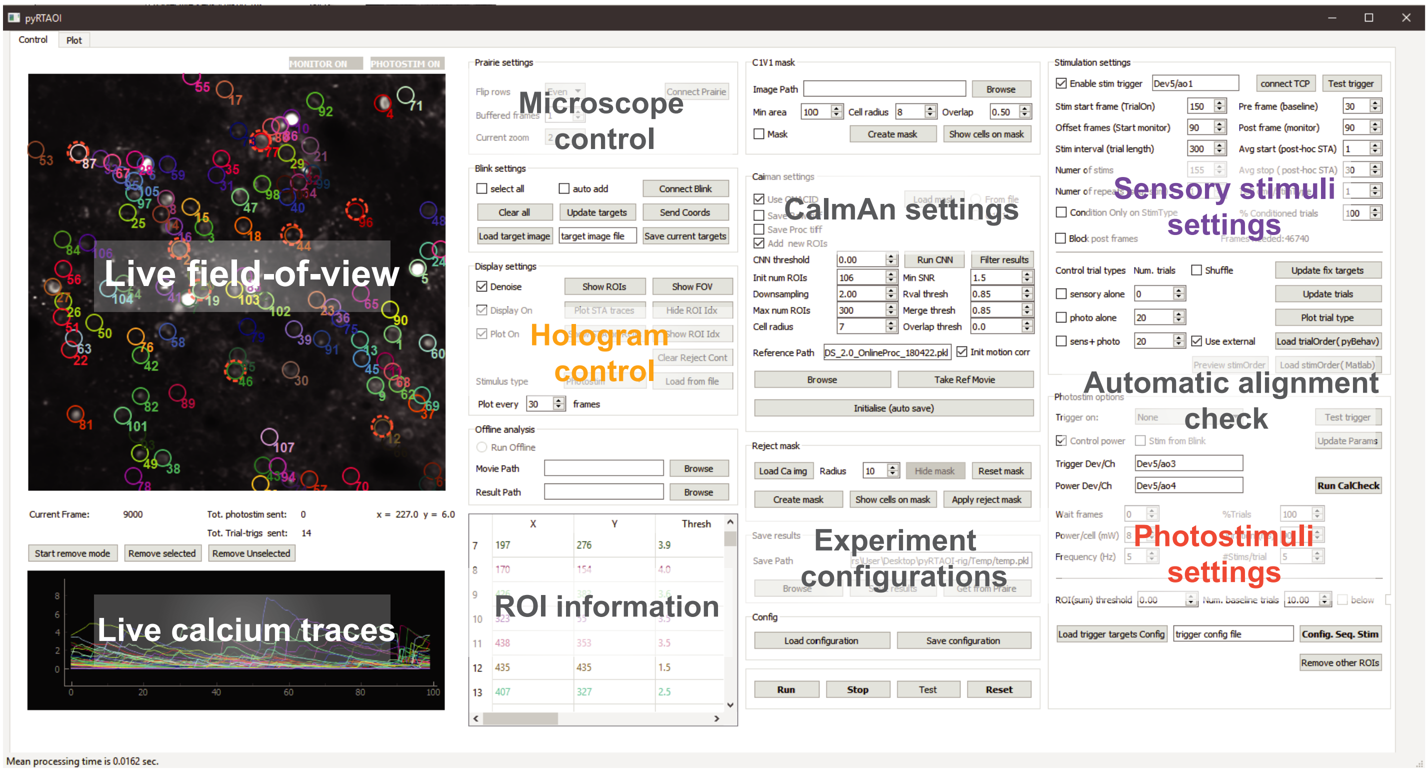

B

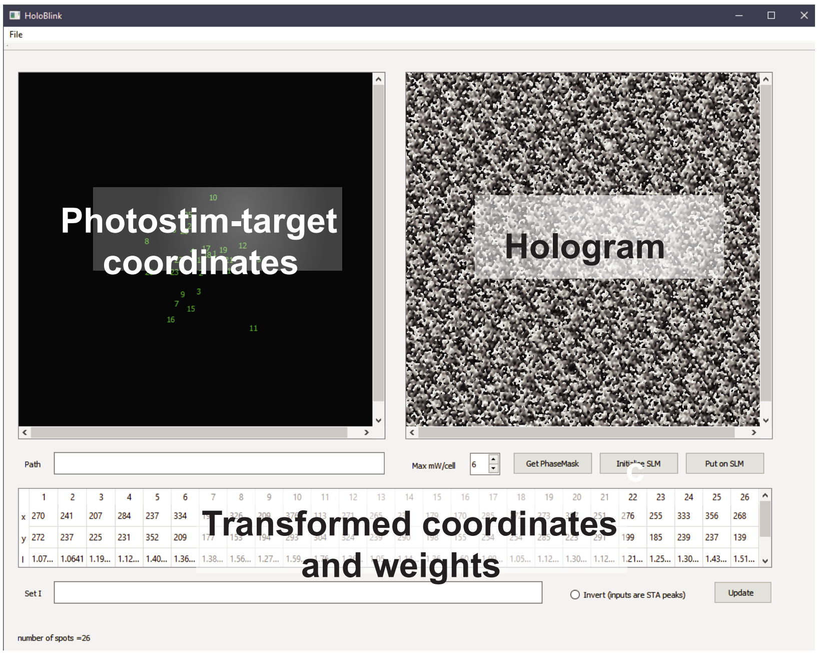

C

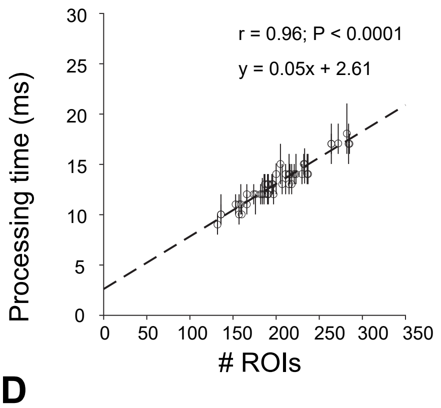

D

Steps	ms
Read samples	2 - 3
Calcium signal extraction	3 - 18
Hologram computation (optional)	~ 15
Hologram display (optional)	~ 13
Trigger spiral photostimulation	<1

Figure S1: Closed-loop all-optical software toolkit, related to Figure 1

(A) The Python-based real-time all-optical interface (pyRTAOI). The key functional modules are labelled, including real-time display of the denoised field-of-view, extracted calcium signal traces, microscope imaging control, photostimulation pattern, power and duration control, access to CalmAn parameter settings, sensory stimulus delivery control, automatic checking of the photostimulation and imaging beam alignment and shortcuts to experiment protocol configurations. (B) The hologram and SLM control interface. An arbitrary group of cells can be stimulated with a two-photon holographic pattern on demand. Photostimulation target locations, weights and hologram to display on the SLM are displayed in the GUI as labelled. (C) The processing time per frame is positively correlated with the number of regions-of-interest (ROIs) in the field of view. Open circles are medians; vertical lines are quartile range for individual sessions. n = 44 sessions, 7 mice. (D) Time budget for each step.

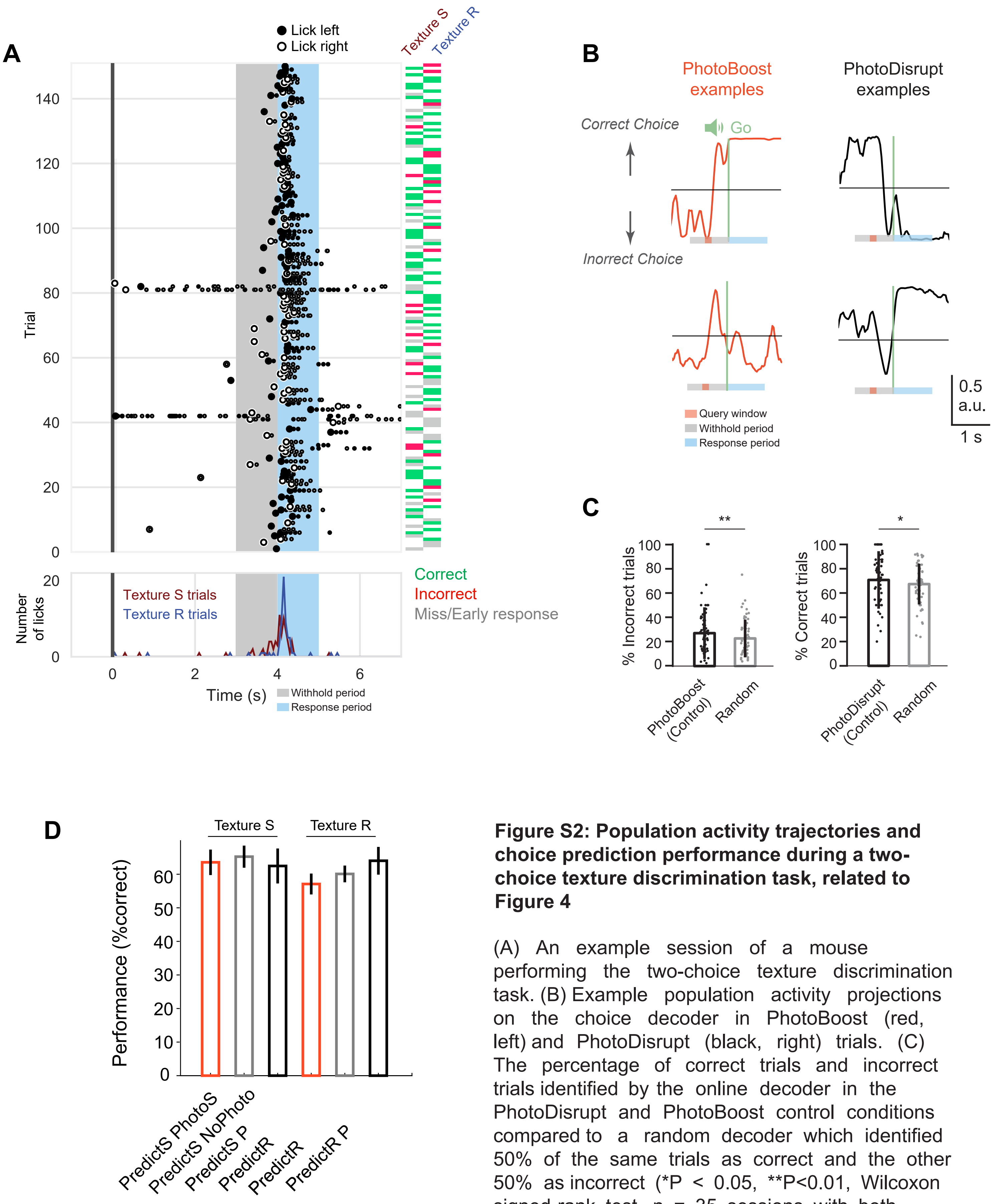

Figure S2: Population activity trajectories and choice prediction performance during a two-choice texture discrimination task, related to Figure 4

(A) An example session of a mouse performing the two-choice texture discrimination task. (B) Example population activity projections on the choice decoder in PhotoBoost (red, left) and PhotoDisrupt (black, right) trials. (C) The percentage of correct trials and incorrect trials identified by the online decoder in the PhotoDisrupt and PhotoBoost control conditions compared to a random decoder which identified 50% of the same trials as correct and the other 50% as incorrect (*P < 0.05, **P<0.01, Wilcoxon signed-rank test. n = 35 sessions with both conditions, 7 mice). (D) Task performance in different trial types predicted by an offline decoder based on the neural activity preceding the photostimulation. S, Smooth; R, Rough.

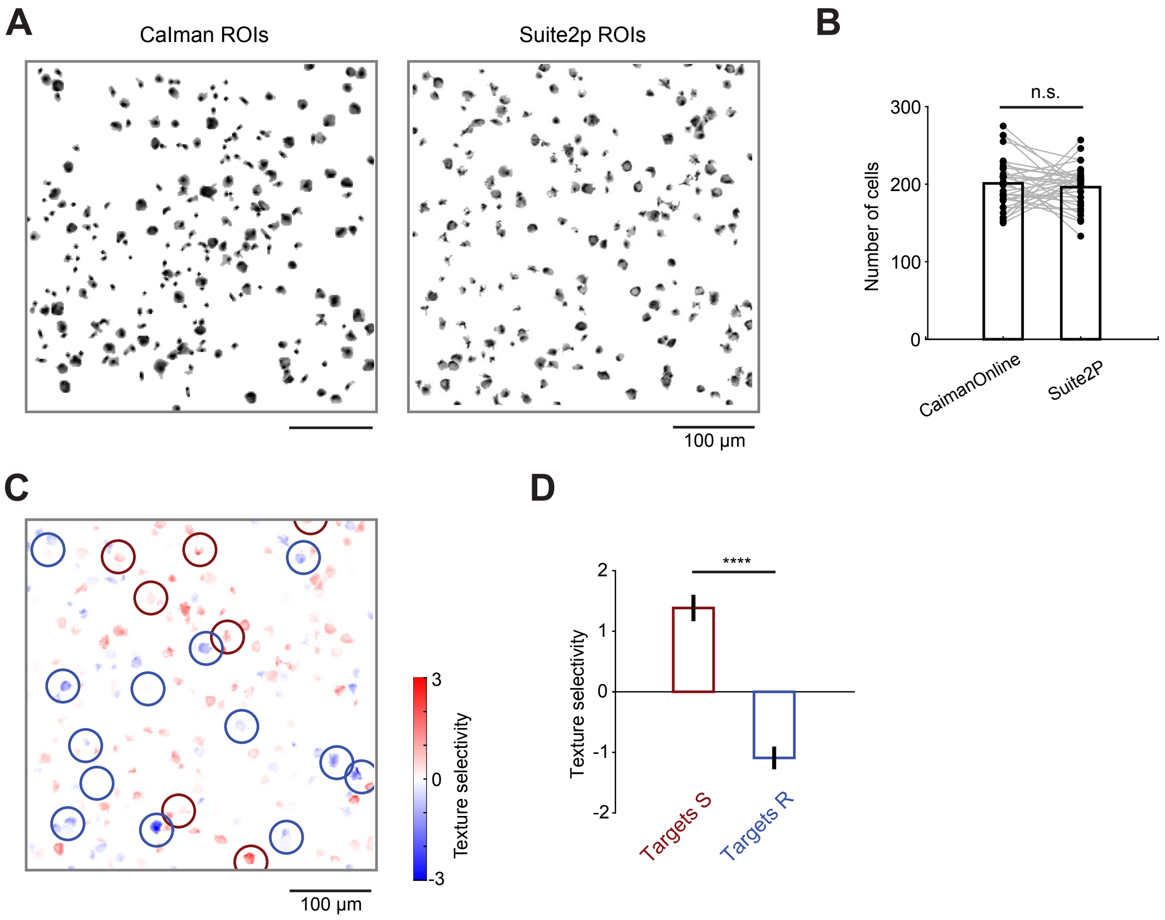

Figure S3: Comparison of online and offline calcium image processing results, related to Figure 4

(A) An example field-of-view (same as in Figure 4) showing the cells detected online by CaImAn and the cells detected offline by Suite2P. Red and blue circles mark the target positions for Texture S and Texture R preferring neurons. (B) Number of cells detected online by CaImAn, filtered by a CNN classifier, and post-hoc by Suite2P, filtered manually (Wilcoxon signed-rank test, n = 33 FOVs). (C) Offline computed texture selectivity of ROIs in the example FOV (positive means Texture S preferring, negative Texture R preferring). (D) Texture selectivity of target ensembles as computed offline (****P < 0.0001, Wilcoxon rank sum test, n = 29 ensembles for Targets S and 29 ensembles for Targets R, 7 mice).

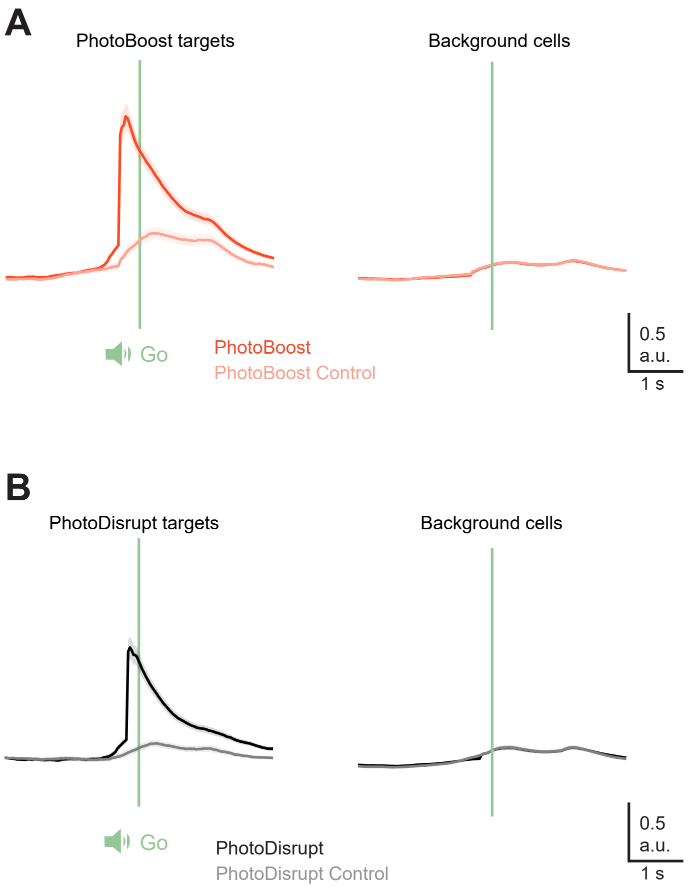

Figure S4: Response to photostimulation in targets and background cells, related to Figure 4

(A) PhotoBoost target responses (left) and background cell responses (right) in the PhotoBoost trials and PhotoBoost Control trials (n = 50 conditions, 7 mice). (B) Same as (A) but for PhotoDisrupt and PhotoDisrupt Control trials (n = 47 conditions, 7 mice).

Criteria	Zhang et al, *Nat. Methods,* 2018 'RTAOI'	Sheng et al, *Front. Cell. Neurosci.*, 2022 'ORCA'	Bowen et al, *eNeuro*, 2024 'NeuroART'	This paper 'pyRTAOI'
Number of cells for activity readout	3	Demonstrated 40	~150	300+
Motion correction	Yes	Yes	Yes	Yes
Online cell detection	No	Yes	Yes	Yes
Neuropil subtraction	No	No	Yes	Yes
Calcium signal denoising	Basic	Model-based	Model-based	Model-based
Photostimulation target selection	Pre-defined	Online updated	*In vitro* demonstration only	Pre-defined or online updated
Online hologram computation	No	Yes	Yes	Yes
Photostimulation power/frequency control	No	No	No	Yes
Microscope imaging control	No	No	No	Yes
Automated alignment check	No	No	No	Yes
Neural activity & Photostimulation hologram display	No	Partially	Yes	Yes
Program language	VB.NET, C++	MATLAB	MATLAB	Python, C++
Closed-loop control during goal-directed behavior	No	No	No	Yes

Table S1: Performance advantage of the new closed-loop all-optical system, related to Figure 1
